# Associations Between REM Sleep-like Posture Expression and Cognitive Flexibility in 2-Month-Old Japanese Black Calves

**DOI:** 10.3390/ani15233438

**Published:** 2025-11-28

**Authors:** Sita Liu, Norihiro Fujita, Takako Sasaki, Takashi Chiba, Shinsuke Konno, Sanggun Roh, Michiru Fukasawa

**Affiliations:** 1 Graduate School of Agricultural Science, Tohoku University, Sendai 980-8572, Japan; norihiro.fujita.b4@tohoku.ac.jp (N.F.); takako.sasaki.a3@tohoku.ac.jp (T.S.); takashi.chiba.e3@tohoku.ac.jp (T.C.); sanggun.roh@tohoku.ac.jp (S.R.); 2 Graduate School of Agricultural Science, Yamagata University, Tsuruoka 997-8555, Japan; skonno@tds1.tr.yamagata-u.ac.jp; 3 Graduate School of Food, Agricultural and Environmental Sciences, Miyagi University, Sendai 982-0215, Japan

**Keywords:** sleep behavior, REM sleep-like posture, cognitive flexibility, associative learning, reversal learning, calf welfare

## Abstract

Sleep supports brain development and adaptability, yet its relationship with cognitive performance in farm animals remains unclear. This study examined the association between a behavioral indicator of rapid eye movement (REM) sleep, the REM sleep-like posture (RSLP), and cognitive ability in Japanese Black calves. Twenty two-month-old calves (11 males and 9 females; 88.0 ± 11.0 kg) were continuously video-recorded for 48 h to quantify daily RSLP time, frequency, and bout duration. Calves then performed associative and reversal learning tasks using color cues paired with rewarding (sweet) or aversive (acidic) fluids. Daily RSLP time, frequency, and bout duration were 220.9 ± 67.3 min/day, 34.9 ± 10.0 bouts/day, and 6.8 ± 2.1 min/bout. Fifteen calves (7 males and 8 females) reached the associative learning criterion, while five (1 female and 4 males) did not. No association was found with associative learning, whereas calves with longer daily RSLP time required fewer rounds to reach the reversal learning criterion (r = −0.56, *p* = 0.030), indicating greater cognitive flexibility. These findings suggest that longer REM-related sleep may support adaptability in calves. Monitoring RSLP could provide a non-invasive indicator of welfare and cognitive capacity, supporting precision management to improve comfort and productivity of calves.

## Simple Summary

Sleep plays an important role in brain development and adaptability in animals. However, its link to cognitive ability in livestock remains unclear. This study investigated a behavioral indicator of rapid eye movement (REM) sleep called the REM sleep-like posture (RSLP) in 20 Japanese Black calves (11 males and 9 females). Calves were continuously video-recorded for 48 h to measure RSLP expression and then completed cognitive tasks: associative learning, which tested their ability to form a simple rule (15 calves learned, including 7 males and 8 females), and reversal learning, which tested their ability to adapt when the rule changed. Calves that spent more time in RSLP reached the reversal-learning criterion faster, suggesting that REM-related sleep supports cognitive flexibility. These results indicate that observing this sleep-related behavior may help identify individual differences in adaptability and welfare among calves. With recent advances in farm video systems and AI-based behavior detection, RSLP could be automatically used as a practical tool to identify calves that may benefit from tailored management to enhance comfort, resilience, and productivity.

## 1. Introduction

Sleep is one of the most fundamental behaviors in animals and occupies a large proportion of mammalian life [1], supporting essential processes such as energy restoration, neural repair, homeostatic regulation, and emotional balance, and playing a central role in physiological stability, brain development, and behavioral adaptability [1,2,3,4,5]. Adequate sleep is especially important in early life, when physiological and neurological systems develop rapidly [1,3,6]. Young mammals typically exhibit longer total sleep duration and a greater proportion of rapid eye movement (REM) sleep compared with adults, both of which decline with age [7,8]. In calves, Fukasawa [2] reported that this sleep-related posture is most common shortly after birth. Its expression gradually decreases as calves grow older, a pattern that aligns with developmental trends observed in many other species. This prolonged REM activity during early life is believed to facilitate neural plasticity and support the establishment of cognitive and emotional regulation [9]. In livestock, adequate sleep and rest contribute directly to both welfare and productivity. Disturbance or deprivation can activate stress responses and impair growth and immune function, whereas sufficient rest supports recovery, adaptability, and overall resilience [7,10,11]. Despite its importance, the link between REM sleep-like behavior and cognitive ability remains poorly understood in farm animals.

Sleep and learning ability have long been considered to be closely linked [5]. Sleep contributes to memory consolidation, neural plasticity, and emotional regulation [2,3,4,5,12,13,14], which support cognitive performance. In farm animals, cognitive ability determines how well individuals cope with challenges [15,16,17]. Livestock species, including donkeys, horses, goats, pigs, and cattle, possess considerable cognitive capacities [18,19,20,21]. In domesticated environments, where conditions are typically more predictable, animals readily form cue–outcome associations through associative learning, such as Pavlovian conditioning [22]. For instance, cattle can associate a specific time or cue with feeding events or link an auditory signal with a mild electric stimulus, enabling the use of virtual fencing as an ethical management tool [23]. Associative learning tasks, therefore, assess how animals form stable cue–outcome connections under routine farm conditions. Cognitive flexibility, defined as the capacity to modify learned responses when conditions change, is critical for adaptation [16,17]. This ability is often evaluated through reversal learning tasks, in which animals first learn a reward-based rule and later must inhibit the previously reinforced response when the rule is reversed [24,25]. Reversal learning paradigms have been successfully applied to calves and are recognized as reliable indicators of flexibility and adaptive coping in farm animals [26]. Adaptive flexibility also underlies behavioral responses to common management challenges, such as weaning, regrouping, and handling [15]. Recent evidence shows that personality traits such as boldness influence habituation and learning in dairy cattle [27], suggesting that individual temperament may also modulate the association between sleep-related behavior and cognitive adaptability. Calves can successfully perform simple color discrimination and reversal learning tasks [26], making these paradigms suitable tools to assess both associative learning ability and cognitive flexibility. By examining the relationship between sleep and these cognitive measures, this study may help clarify how early-life sleep is linked to behavioral adaptability and welfare in farm animals [15,16,17].

Sleep-like behavior provides a non-invasive behavioral measure of sleep. Sleep in livestock is commonly assessed through video observation, accelerometers, or electroencephalogram (EEG) recordings [6,25,28]. Although EEG provides precise detection of sleep stages, it requires head-mounted sensors that are secured using straps or ropes around the body, making it clearly intrusive and likely to alter natural behavior [6,28]. Accelerometers involve a smaller attachment area and are therefore less intrusive, but they still require devices to be fixed to the animal and can only capture a limited range of postures, making them unsuitable for distinguishing specific sleep-related behaviors [29]. Therefore, video-based behavioral observation is preferred as a fully non-intrusive method for assessing natural sleep-related behaviors under farm conditions.

Two postures have been linked to sleep in calves: “resting with head lifted up still,” corresponding to non-rapid eye movement (NREM) sleep, and “resting with neck relaxed”, corresponding to rapid eye movement (REM) sleep [6,30]. During the latter posture, calves lie laterally or sternal with the neck and chin resting on the ground and show reduced responsiveness to external stimuli, consistent with the muscle atonia typical of REM sleep. When this posture lasts longer than 30 s, it identifies 61 ± 7% of EEG-verified REM sleep episodes with approximately 90% specificity (κ = 0.31–0.55, *p* < 0.001) [6,31]. Although “resting with head lifted up still” has been validated as an indicator of NREM sleep [6,31], its start and end points are difficult to distinguish, which may be easily confused with drowsiness or quiet wakefulness. Consequently, the present study focuses on the more distinct REM-related posture, defined as the REM sleep-like posture (RSLP). Although RSLP does not indicate REM sleep with 100% accuracy, it still enables continuous, long-term observation without physical restraint or equipment, providing a practical and ethical proxy for REM-related sleep [6,30,32,33].

Therefore, this study aimed to explore the potential association between sleep expression and cognitive performance, particularly associative learning ability and cognitive flexibility, in Japanese Black calves. To our knowledge, no livestock study has linked naturally occurring REM sleep-like behavioral posture with associative and reversal learning performance in young ruminants. We hypothesize that calves exhibiting longer RSLP, reflecting more consolidated REM-related sleep expression, would show better associative learning and adaptive performance, indicating enhanced cognitive ability and flexibility.

## 2. Materials and Methods

### 2.1. Ethical Approval

All experimental procedures were approved by the Tohoku University Animal Experiment Committee (Approval No. 2023-002-02). The study was conducted at the Kawatabi Field Science Centre, Tohoku University (Osaki, Miyagi, Japan). Behavioral data were collected from July 2023 to May 2024, covering both summer and winter seasons in Kawatabi Field Science Centre.

### 2.2. Housing and Animals

The study included twenty clinically healthy Japanese Black calves (11 males and 9 females, weight 88.0 ± 11.0 kg), all aged two months at the beginning of the experiment. Calves at approximately two months of age were selected because this age group was available at the experimental field and represents a stable period after dam separation (around 0.5 months old) and adaptation to artificial rearing. Younger calves (around one month) may still be affected by separation stress and early environmental changes, whereas older calves begin weaning and show changes in resting and feeding behavior. Each calf was housed individually in a 1.7 × 1.7 m pen that allowed visual and auditory contact with neighboring calves. Pens were bedded with sawdust and cleaned twice daily. Calves were fed milk, hay, and milk replacer twice a day to meet nutritional requirements. No enrichment materials were provided in the pens to avoid interference with video recording. The barn followed a natural light–dark rhythm, supplemented by ceiling lights used during routine work periods (09:00–12:00 and 15:00–17:30). Ambient temperature was confirmed not to be related to RSLP expression or cognitive performance; the raw data are available in the Supplementary Materials. Relative humidity was not recorded; however, the year-long study period, which covered both summer and winter seasons, increased the generalizability of the research. Background noise was minimal, with only occasional sounds from machinery operating near the shelter (approximately 1–4 times per month). Daily health checks assessed appetite, fecal condition, and general behavior, with body temperature measured only if abnormalities were observed to avoid unnecessary stimulation. All calves remained clinically healthy, with no signs of diarrhea, respiratory disease, or other illness observed before or during behavioral observation.

### 2.3. RSLP Observation

RSLP was monitored using ceiling-mounted cameras (Qwatch TS-NA230WP, I-O Data Device, Inc., Kanazawa, Japan) positioned above the center of each pen at a height of 3.3 m. Video data were continuously recorded and stored on external hard drives (HDJA-UTN24/LDB, I-O Data Device, Inc., Kanazawa, Japan). Each camera recorded continuously in Full HD (1920 × 1080 pixels) at 30 frames per second, and the field of view covered the entire pen area without blind spots. The lens was oriented perpendicular to the floor, and no occlusion of calves was observed during recording. Each calf was recorded continuously for 48 h at 2 months of age using two successive 24 h periods. RSLP was defined as a lying posture with the “resting with neck relaxed” and no visible movement for at least 30 s [6]. From each 24 h recording, total RSLP time, number of bouts, and mean bout duration were calculated. Behavioral coding was conducted continuously by a single trained observer (S.L.) using consistent definitions throughout all analyses to ensure reliability. Videos were played at 4× speed for standing and 2× speed for lying postures using VLC Media Player Version 3.0.21 (VideoLAN, Paris, France) for annotation.

### 2.4. Associative Learning Test and Reversal Learning Test

The calves underwent two cognitive tests: the associative learning test and the reversal learning test. The associative learning test was initiated on the day following completion of the 48 h RSLP video recording, while the reversal learning test was conducted in the next round after calves met the learning criterion. All cognitive tests were performed in the calves’ home pens to minimize stress and avoid the need for adaptation to a new environment. The associative learning test was initiated within 24 h after the completion of RSLP recording to maintain temporal continuity between sleep observation and cognitive testing. All tests were conducted in two fixed daily time slots (08:00 a.m. and 2:00 p.m.) to minimize potential circadian or daytime effects on performance. The testing order was determined based on calves’ birth order (tag numbers) and remained consistent across testing days. The light cycle and other environmental conditions were consistent with those described in Section 2.2 and remained stable throughout the one-year study period.

**Figure 1:**
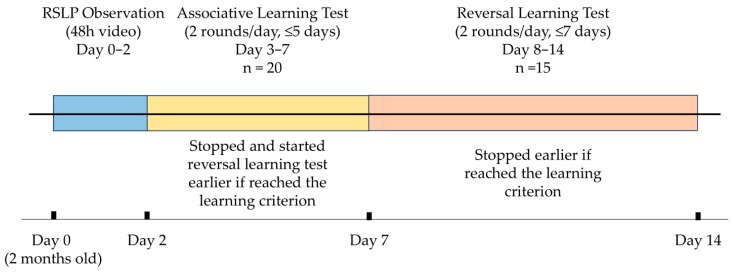
Experimental schedule for REM sleep-like posture (RSLP) observation and cognitive testing in Japanese Black calves. Twenty calves were observed continuously for 48 h (Days 0–2) to record RSLP behavior. The associative learning test was then conducted twice daily for up to five days (Days 3–7), followed by the reversal learning test, which was conducted twice daily for up to seven days (Days 8–14). Fifteen calves (*n* = 15) that met the learning criterion (average accuracy ≥ 90% across two consecutive rounds) progressed to the reversal-learning phase. Each test was stopped earlier if a calf reached the learning criterion.

The experimental schedule is illustrated in Figure 1. RSLP observation was conducted on the exact day calves reached two months of age, with continuous video recording for 48 h (Day 0–2). The associative learning test began on the following day (Day 3) and was conducted twice daily (2 rounds per day) for a maximum of five days (Day 3–7). Calves were considered to have reached the learning criterion when their average accuracy rate across two consecutive rounds (10 trials per round) was ≥90%. Once this criterion was achieved, the associative learning test was terminated, and the calf proceeded to the reversal learning test in the following round. The reversal learning test was initiated in the round following the learning criterion and continued for a maximum of 14 rounds across up to seven days. As in the associative phase, testing was terminated earlier if calves reached the learning criterion before the maximum duration of seven days.

#### 2.4.1. Associative Learning Test

The associative learning test was designed to train calves to associate visual color cues with either a positive (rewarding) or negative (aversive) fluid. Black and white buckets were selected because calves can discriminate between these colors [34,35] and use them effectively in learning tasks [26]. Sweet and acidic tastes were used as positive and aversive stimuli, respectively, since cattle strongly prefer sucrose concentrations above 2.5% and avoid acetic acid concentrations above 0.08% [36]. Accordingly, a 5% sucrose solution served as the reward, and a 0.5% acetic acid solution served as the aversive stimulus. These concentrations created a clear but non-noxious hedonic contrast suitable for learning [24,37]. Milk was not used to avoid nutritional confounding and satiety effects.

Each calf was presented with two buckets—one black and one white—each containing either the sweet or the acidic transparent solution. The color–fluid mapping (sweet vs. aversive) was randomized individually for each calf using a random-number formula in Microsoft Excel, and allocation was independent of sex. The color–fluid combination remained constant within each 10-trial round, while the left–right side placement was randomized such that the rewarded side did not repeat more than three consecutive times. This design prevented calves from learning a fixed spatial order and ensured that their responses reflected the color–reward association rather than side bias.

A maximum of 10 rounds was conducted, each consisting of 10 trials. Two rounds were performed daily at 08:00 a.m. and 2:00 p.m. A calf was considered to have learned the association when it chose the rewarded color in at least 90% of trials across two consecutive rounds. The number of rounds required to reach this criterion was recorded as associative learning rounds (ALR). Calves that reached this criterion were advanced to the reversal learning test, while those failing to do so within 10 rounds were classified as not learned.

To protect calf welfare, the volume offered per round was limited to 0.75 L, as too much sucrose may lead to diarrhea and overly acidic solutions may cause irritation. Both buckets were removed once the calf had drunk or leaked fluid for about 5 s. If a calf failed to drink from either bucket within 2 min, both buckets were removed and the next trial began. Tests were performed in the home pen without additional handling. The experimenter (S.L.) placed and removed the buckets directly in front of the pen and then waited at a distance of approximately 1 m for the calf’s response. The same experimenter carried out feeding and cleaning three times per week, so calves were familiar with the person. Inter-trial intervals ranged from 5 to 30 s, beginning once the calf’s head no longer obstructed the placement area. Buckets were rinsed thoroughly with fresh water three times after each round to minimize residual scent cues. Testing was conducted 1–2 h before regular feeding to maintain motivation and avoid disturbance from daily routines.

#### 2.4.2. Reversal Learning Test

The reversal learning test was conducted in the next round after the calf achieved the learning criterion in the associative task. This test evaluated cognitive flexibility by assessing how quickly calves adapted to reversed color–fluid associations. The reward and aversive solutions were swapped between the black and white buckets, while the overall structure, randomization procedure, and trial schedule remained identical to the associative learning test. The reversal phase continued for up to 14 rounds (7 days). A calf was considered to have successfully learned the reversal when it achieved ≥90% correct responses in two consecutive rounds. The number of rounds required to reach this criterion was recorded as reversal learning rounds (RLR).

### 2.5. Statistical Analysis

All statistical analyses were performed using Minitab^®^ Version 21.4 (64-bit), with the significance level set at *p* < 0.05. Data from all 20 calves were included in the analyses. Because all calves were tested at fixed times (08:00 a.m. and 2:00 p.m.), potential time-of-day effects were controlled experimentally and were not included as an analytical factor. Behavioral observations were conducted for two consecutive days per individual. Therefore, to minimize day-to-day variation, daily values were averaged to obtain a representative mean for each calf, which was then used in all subsequent analyses.

To compare RSLP expression between calves that learned the associative learning training and those that did not, Welch’s two-sample *t*-tests for unequal variances were applied. The study included 20 Japanese Black calves (11 males and 9 females). Among the five calves that did not learn the associative learning task, one was male and four were female. To examine whether the sex distribution among calves that did not learn (four male and one female calves) deviated from chance, an exact binomial test was conducted using a 50% expected probability for each sex. A post hoc power check was performed to evaluate the sensitivity of the comparison.

The normality of all variables was first assessed using the Shapiro–Wilk test. For calves that successfully learned (*n* = 15), relationships between RSLP expression and cognitive performance (ALR and RLR) were analyzed using correlation tests. Pearson’s correlation was applied for normally distributed variables, and Spearman’s rank correlation for non-normal variables. The interpretation of correlation strength followed Cohen (1988) [38], where |r| = 0.10–0.29 indicates a small, |r| = 0.30–0.49 a moderate, and |r| ≥ 0.50 a strong correlation. To control for potential sex effects, partial correlations controlling for sex were additionally performed as sensitivity analyses using the same correlation method as in the main analysis, to confirm that the observed associations were not influenced by sex.

## 3. Results

Across all calves (*n* = 20; 11 males, 9 females), mean (±SD) values for the average daily RSLP time, frequency, and bout duration were 220.9 ± 67.3 min/day, 34.9 ± 10.0 bouts/day, and 6.8 ± 2.1 min/bout, respectively. Fifteen calves learned the associative learning task (7 males, 8 females), and five did not (4 males, 1 female). The overall mean number of associative and reversal rounds was 6.7 ± 2.3 and 5.4 ± 2.5, respectively (*n* = 15).

Group comparisons of daily RSLP time, frequency, and bout duration are summarized in Table 1. The learned (*n* = 15) and not-learned (*n* = 5) calves showed similar ranges across all RSLP variables, and no clear group differences were apparent from the descriptive values. Four of the five not-learned calves were males, resulting in a sex imbalance between groups. As the not-learned group was very small, a post hoc power check based on the observed effect sizes indicated low statistical power (0.05–0.16). Therefore, no statistical inferences were drawn from these data.

**Figure 2:**
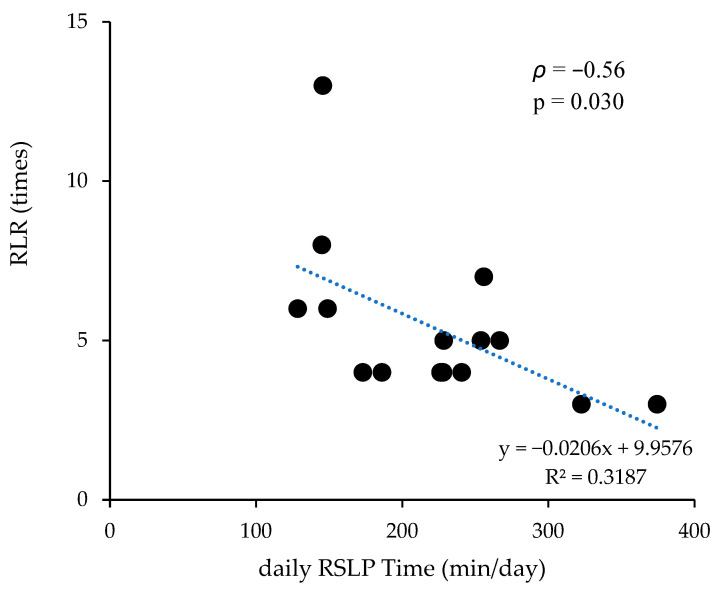
Correlation between daily time spent in rapid eye movement (REM) sleep-like posture (RSLP) and reversal learning performance (reversal learning rounds, RLR) in Japanese Black calves (*ρ* = −0.56, *p* = 0.030, *n* = 15). Each point represents one calf; the line shows the linear regression trend.

All RSLP variables (daily time, frequency, and bout duration) and associative learning rounds followed a normal distribution (*p* > 0.05), whereas reversal learning rounds did not (*p* < 0.05). Correlation results are summarized in Table 2. ALR and RLR were not correlated (*ρ* = 0.02, *p* = 0.958). ALR showed a moderate negative correlation with daily RSLP time (*r* = −0.34, *p* = 0.218) and small negative correlations with RSLP frequency (*r* = −0.15, *p* = 0.586) and RSLP bout duration (*r* = −0.17, *p* = 0.545). RLR showed a strong negative correlation with daily RSLP time (*ρ* = −0.56, *p* = 0.030, *n* = 15; Figure 2). Partial correlations controlling for sex also showed a strong negative association (*ρ* = −0.52, *p* = 0.046). RLR were moderately and negatively correlated with RSLP frequency (*ρ* = −0.34, *p* = 0.217) and RSLP bout duration (*ρ* = −0.41, *p* = 0.131). The interpretation of correlation strength followed Cohen (1988) [38].

## 4. Discussion

This study examined the expression of REM sleep-like posture (RSLP) in Japanese Black calves and its association with early-life cognitive performance. RSLP expression did not differ between calves that learned the associative task and those that did not, but the small size of the not-learned group and the resulting low statistical power limit the reliability of this comparison. No correlation was found with associative learning, whereas calves that showed longer daily RSLP time tended to perform better in the reversal learning.

The distribution and characteristics of RSLP observed in this study were consistent with previously reported sleep parameters in calves and fell within the expected biological range. Although the posture-based approach produced fewer and longer bouts (REM sleep = 162 ± 28 min/day; total sleep frequency = 50 ± 22 bouts/day; total sleep bout duration = 5 ± 2 min/bout), the present RSLP time was approximately 60% longer, frequency about 30% lower, and bout duration about 35% longer than EEG-based estimates [6]. These differences likely reflect methodological and developmental factors: the EEG study recorded for only 20 h, used a smaller sample of slightly older calves (*n* = 6, 3 months old), an age at which total sleep and REM proportions decline [7,8], quantified frequency and bout duration across both REM and NREM (total sleep) rather than REM alone, and used EEG equipment that may have influenced natural sleep expression [6]. Compared with accelerometer-based assessments (REM sleep ≈ 110–150 min/day; frequency ≈ 12–13 bouts/day; bout duration ≈ 9–12 min/bout) [2], the present RSLP durations were approximately 60–100% higher, frequencies substantially higher, and bout durations 30–40% shorter. These differences reflect the posture definitions and duration criteria used. Fukasawa et al. [1] focused on a narrower posture, “lying with the neck bent and the head resting on the flank”, and counted only bouts lasting at least 2 min [29]. They analyzed only bouts longer than 2 min, whereas the present study included bouts exceeding 30 s. In contrast, our study applied the broader “resting with neck relaxed” posture and a 30 s threshold, which has been validated to correspond to most EEG-confirmed REM episodes [6,31]. This shorter threshold allows brief REM-like bouts to be captured, although it may occasionally include quiet wakefulness, whereas the 2 min criterion offers higher specificity but may underestimate REM-related sleep by excluding shorter, physiologically relevant episodes. Additionally, Fukasawa et al. [39] reported significant differences in daily sleep time, frequency, and bout duration of sleep among 12 commercial dairy farms, despite similar management conditions. Given the large standard deviations in both the present dataset and earlier studies, percentage contrasts should be considered approximate. Overall, RSLP offers a practical and non-intrusive indicator of REM-related activity, although its physiological specificity remains imperfect, highlighting the need for further validation studies combining behavioral observation with EEG across ages and housing systems.

Group comparisons between calves that learned the task and those that did not showed similar ranges for daily RSLP time, frequency, and bout duration. The not-learned group was small, with only five calves, and four of them were male. This limited the ability to examine group patterns. A post hoc power check showed low sensitivity for detecting small differences, meaning that small patterns may not have been detectable. Studies with larger and more balanced groups would allow clearer evaluation of whether variation in RSLP expression relates to learning performance or whether such associations appear only under specific developmental or environmental conditions.

No strong association was found between RSLP expression and ALR. This diverges from findings in humans and laboratory animals linking sleep with memory consolidation and basic learning [4,40,41]. One explanation is that associative tasks may rely on striatum-based procedural systems that operate relatively independently of REM sleep. The striatum supports habit-like, stimulus–response learning, which relies on procedural processes that do not depend strongly on sleep. In contrast, the hippocampus plays a key role in forming new associations and organizing memories, and this region is more sensitive to sleep loss. For example, Hagewoud et al. [42] showed that sleep-deprived mice maintained spatial learning by shifting from hippocampus-dependent to striatal strategies. Another consideration is the timing of sleep relative to training: in humans, improvements in associative memory occur when sleep follows training, whereas no benefit is seen when sleep is restricted to daytime or deprived overnight [43]. Although our round-level analyses did not reveal a clear relationship between RSLP and associative learning, this may reflect a performance-ceiling constraint. Because calves were trained to a ≥90% criterion and learning was summarized by rounds, modest sleep-related effects could have been masked. The individual learning curves (Supplementary Figures S1 and S2) illustrate substantial inter-calf variation in acquisition rate. Future studies should record trial-level data and apply generalized linear mixed-model approaches to test for subtler relationships between sleep patterns and learning dynamics.

Daily RSLP time was negatively correlated with RLR, indicating that calves expressing longer daily RSLP time adapted more quickly when the task rules reversed. This supports evidence that REM sleep contributes to behavioral flexibility, emotional regulation, and the integration of new information [42,44,45]. During early life, REM plays a central role in shaping neural circuits underlying executive functions, particularly cognitive flexibility. REM promotes synaptic organization in the medial prefrontal cortex, and disruption during sensitive developmental stages can impair synaptic maturation and long-term adaptability [46,47]. REM provides a distinct neurophysiological state that reduces aminergic interference, reorganizes weaker associative links, and transforms episodic experiences into generalized representations through hippocampal reactivation [14,48]. Within the RSLP expressions, daily time showed the strongest association with reversal learning, whereas frequency and bout duration were only moderately related. This likely reflects differences in how accurately the three measures represent RSLP and how relevant each is to learning performance. Daily time represents the overall amount of REM-related processing across the observation period, which is most directly linked to neural plasticity and cognitive flexibility [46,47]. In contrast, frequency and bout duration are more easily influenced by short arousals, husbandry routines, posture adjustments, and the 30 s threshold used to define bouts, all of which increase variability and weaken correlations. The limited sample size (*n* = 15) also reduced the power to detect moderate effects. These factors may explain why only RSLP time showed a strong correlation with RLR, whereas frequency and bout duration were more weakly related. Although the association between RSLP and reversal learning suggests a link between REM-related sleep and flexibility, causality cannot be revealed from correlational data. Studies that manipulate sleep opportunity or vary the timing of testing relative to sleep would help clarify directionality. In this context, calves with longer RSLP durations may benefit from enhanced prefrontal processing and adaptive capacity, but this interpretation remains tentative and requires further physiological validation.

Although sex was not a primary focus of this study, it was included as a control factor to account for potential variation. Four of the five calves that failed to learn the associative task were male, but the small size of this group limits any further consideration of sex-related patterns. No significant sex effects were detected for RSLP variables or learning performance, indicating that the observed associations were not driven by sex. Nevertheless, sex-related variation in sleep and cognition has been reported in other species—for example, female mice exhibit longer REM sleep episodes than males under baseline conditions [49], and female dogs show higher NREM spindle density and superior learning performance compared with males [50]. However, it should be noted that no sex-related differences were found among the calves that successfully learned the task. Future studies with larger, sex-balanced samples are needed to confirm whether early-life sleep organization contributes to subtle sex differences in learning and cognitive flexibility in calves.

Learning performance might also be influenced by temperament. Calves differ in exploratory behavior, neophobia, and fearfulness [51,52,53]. These traits can affect how readily they engage with new tasks. More exploratory calves approach more quickly, while more fearful individuals hesitate or avoid [52,53], so some variation in learning may reflect temperament rather than sleep alone. Sleep, temperament, and emotion are closely connected. REM sleep disruption can impair emotional regulation [54], and sleep and cognitive development are linked across species [55]. For example, insufficient sleep in children is associated with poorer academic performance and higher levels of anxiety and attention difficulties [40]. The presence of the experimenter during testing may also have influenced more reactive calves and affected their performance. Because temperament indicators such as latency or refusals were not recorded, future studies should include standardized assessments, such as novel-object or open-field tests, to clarify how reactivity interacts with sleep patterns and learning flexibility.

In addition to these findings, several methodological and behavioral factors may have influenced the results. Both test fluids had a faint odor detectable to humans, and calves may also have sensed this difference. Although olfactory cues could have contributed to discrimination, this does not undermine the purpose of the task, which was to assess associative learning through any consistent cue. Differences in cue salience may also have affected task sensitivity, as spatial cues are generally more salient to cattle than color, and visual cues paired with auditory stimuli can evoke stronger responses, while repeated exposure can lead to habituation [56]. Repeated presentation of the 5% sucrose reward may have reduced motivation over trials, weakening reinforcement and affecting task performance, particularly in calves with lower natural motivation or quicker satiation. These factors, along with individual differences in reward sensitivity, may help explain the variation observed in associative learning. The study also had several limitations, including a small sample size and only a few calves that did not learn the associative task, which reduced statistical power. The work was conducted only on Japanese Black calves, a beef breed that may differ from dairy breeds in temperament, sleep patterns, and growth, which limits how broadly the findings can be applied. Individual housing allowed consistent behavioral measurements but may alter sleep organization and social learning opportunities compared with group housing [26,57,58,59]. The short experimental period prevented assessment of longer-term developmental or welfare outcomes, and future longitudinal studies will be needed to determine whether early-life RSLP predicts later adaptability or productivity. Environmental factors such as temperature, pen design, and routine management may also influence sleep behavior and should be considered in future work to strengthen the ecological validity of RSLP as a welfare indicator.

The association between RSLP and reversal learning has important welfare and management implications. Cognitive flexibility helps animals cope with common challenges, such as weaning, regrouping, handling, and transport [15]. and more flexible calves are likely to adjust more easily and maintain better welfare and productivity. This study provides the first evidence in livestock that a REM-like behavioral marker relates to reversal learning, extending findings from humans and laboratory species. Across animals, sleep loss reduces reversal learning and adaptability, including in magpies [60], dogs [61], and rats [62]. Mechanistic and modelling studies also indicate that REM sleep promotes synaptic reorganization and flexible cognitive processing [63,64]. Recent precision livestock farming tools, such as accelerometers, computer-vision systems, and machine-learning methods, now make continuous on-farm monitoring of sleep and rest behavior feasible [32]. Video-based posture detection is already available for cattle [65]. Barn cameras could be paired with AI-supported pose-estimation models (e.g., T-LEAP) to identify RSLP automatically. Integrating such systems into routine management could help detect calves with unusually low REM-like activity, enabling earlier interventions to reduce stress and improve welfare [16,17,66]. RSLP may therefore function as a practical, non-intrusive marker of adaptive capacity. Calves with low RSLP may benefit from targeted support such as gradual weaning, additional habituation before regrouping or handling, or improved enrichment and resting comfort [15]. In the longer term, including behavioral sleep indicators such as RSLP in welfare assessment and management frameworks could help guide breeding, housing, and rearing strategies that promote comfort, resilience, and sustainable productivity [11,19,66,67].

## 5. Conclusions

This study indicates that calves expressing longer daily time in the REM sleep-like posture (RSLP) tended to perform better in the reversal learning test, suggesting a potential link between REM-related behavior and early-life cognitive flexibility. RSLP may serve as a practical, non-intrusive behavioral indicator of adaptive capacity under farm conditions. It may also complement existing welfare assessment frameworks. The small sample size and the use of a behavioral indicator of REM-related activity mean that further studies are needed to confirm and extend these findings. Studies incorporating EEG-based validation, additional physiological measures, and larger, sex-balanced samples will be important for establishing the robustness and generalizability of RSLP as an early-life marker of welfare and cognitive potential in livestock.

## Figures and Tables

**Table 1:** Comparison of rapid eye movement (REM) sleep-like posture (RSLP) expression between calves that learned (*n* = 15) and those that did not learn (*n* = 5) the associative learning task. Values are presented as mean ± standard deviation (SD). Mean differences are shown with 95% confidence intervals (CI). Two-sample *t*-tests with Welch’s correction were used for all comparisons.

	Learned (Mean ± SD)	Not Learned (Mean ± SD)	Mean Difference (95% CI)	*t* (df)	*p*-Value
daily RSLP time (min/day)	221.5 ± 69.4	218.9 ± 75.9	−2.6 (−96.6, 91.3)	−0.07 (6)	0.947
RSLP frequency (bouts/day)	33.9 ± 10.3	38.0 ± 10.7	4.1 (−9.2, 17.5)	0.76 (6)	0.478
RSLP bout duration (min/bout)	7.13 ± 2.31	5.84 ± 1.21	−1.29 (−3.03, 0.45)	−1.60 (13)	0.134

**Table 2:** Correlations between rapid eye movement (REM) sleep-like posture (RSLP) expression and learning performance (associative learning rounds, ALR; reversal learning rounds, RLR) in calves that successfully completed the associative learning test (*n* = 15). Values are correlation coefficients (*r*, *ρ*) with 95% confidence intervals (CI).

	ALR *r* (95% CI)	*p*-Value	RLR *ρ* (95% CI)	*p*-Value
daily RSLP time (min/day)	−0.34 (−0.73, 0.21)	0.218	−0.56 (−0.85, −0.02)	0.030 *
RSLP frequency (bouts/day)	−0.15 (−0.62, 0.39)	0.586	−0.33 (−0.73, 0.23)	0.217
RSLP bout duration (min/bout)	−0.17 (−0.63, 0.38)	0.545	−0.41 (−0.77, 0.16)	0.131

* Represents significant correlation (*p* < 0.05).

## Data Availability

The original data supporting the findings of this study are included in the Supplementary Material. Further inquiries can be directed to the corresponding authors.

## Supplementary Materials

The following supporting information can be downloaded at: https://www.mdpi.com/article/10.3390/ani15233438/s1, Table S1: Original data of REM sleep-like posture (RSLP) measurements and learning performance of Japanese Black calves. Figure S1: Associative learning accuracy across rounds for individual calves (*n* = 20). Accuracy was calculated as the number of trials in which the calf chose the positive (sweet) fluid divided by 10 trials per round. Each line represents one calf. A maximum of 10 rounds was conducted. Figure S2. Reversal learning accuracy across rounds for individual calves (*n* = 15). Accuracy was calculated as the number of trials in which the calf chose the positive (sweet) fluid divided by 10 trials per round. Each line represents one calf. A maximum of 14 rounds was conducted.
