# Supplementary material for: Associations Between REM Sleep-like Posture Expression and Cognitive Flexibility in 2-Month-Old Japanese Black Calves
Source: Animals (Basel). 2025 Nov 28;15(23):3438. doi: 10.3390/ani15233438 (PMC12691010; doi:10.3390/ani15233438)
Supplement: Supplementary file 1 [file animals-15-03438-s001.zip › Supplementary Figures S1 and S2.pdf]

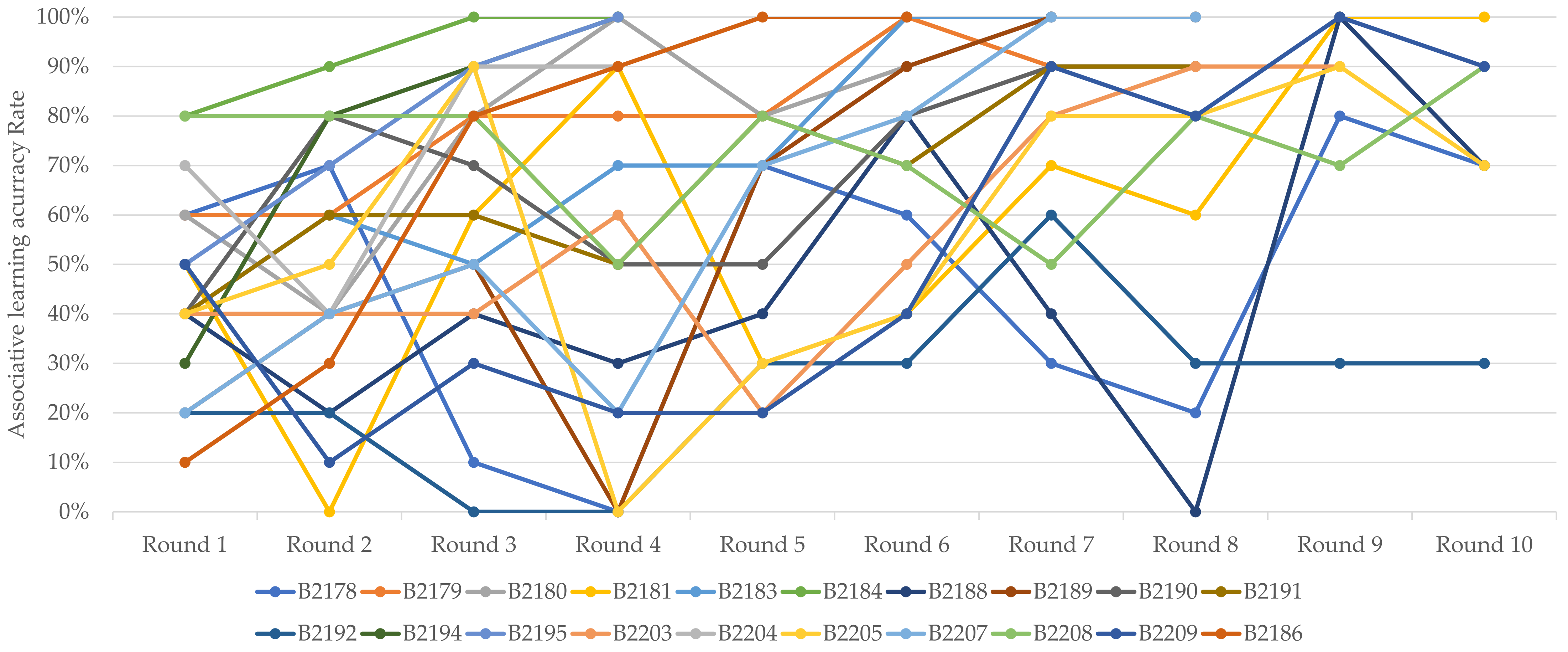

Supplementary Figure S1. Associative learning accuracy across rounds for individual calves (n = 20). Accuracy was calculated as the number of trials in which the calf chose the positive (sweet) fluid divided by 10 trials per round. Each line represents one calf. A maximum of 10 rounds was conducted.

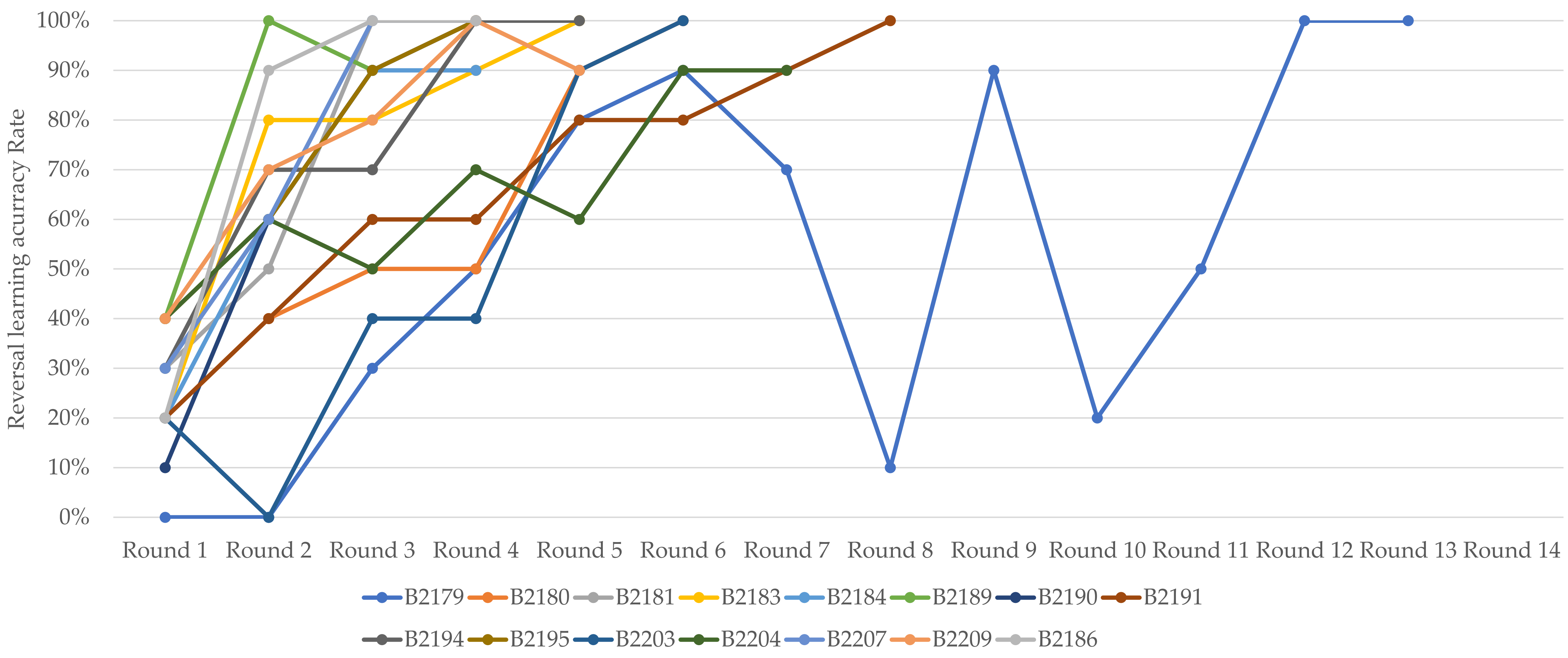

Supplementary Figure S2. Reversal learning accuracy across rounds for individual calves (n = 15). Accuracy was calculated as the number of trials in which the calf chose the positive (sweet) fluid divided by 10 trials per round. Each line represents one calf. A maximum of 14 rounds was conducted.
